# Efficacy and Compliance of a Working Memory Multitasking Task Mobile Intervention for Children With Attention-Deficit/Hyperactivity Disorder: Single-Arm, Pre-Post Pilot Study

**DOI:** 10.2196/70479

**Published:** 2025-10-28

**Authors:** Minyoung Jung, Jimin Woo, Sung Jin Kim, You Bin Lim, Younglae Kim, Dongwon Kang, Jung-sang Min, Bung-Nyun Kim

**Affiliations:** 1 eMotiv Co. Ltd Seoul Republic of Korea; 2 College of Medicine Division of Child and Adolescent Psychiatry, Department of Psychiatry Seoul National University Hospital Seoul Republic of Korea

**Keywords:** attention-deficit/hyperactivity disorder, digital therapeutics, cognitive training, gamification, efficacy, feasibility, multitasking, n-back

## Abstract

**Background:**

Attention-deficit/hyperactivity disorder (ADHD) is a prevalent neurodevelopmental disorder affecting 3%-7% of children globally. Alternative treatments are needed to address the limitations of traditional pharmacotherapy and nonpharmacotherapy, such as drug side effects and substantial time and financial costs. In this light, digital therapeutics for childhood ADHD are emerging as an effective alternative, with the benefits of potentially being free from serious side effects associated with software-based treatments and facilitating easy home use without constraints on time or space.

**Objective:**

This study aims to evaluate whether a 4-week digital treatment program can improve symptoms, problem behaviors, and neurocognitive functions in children with ADHD, independent of medication status, while also gauging participant satisfaction with the program.

**Methods:**

We recruited 22 Korean children aged 6-12 years with a diagnosis of ADHD. During the preintervention visit, we collected data on ADHD symptoms, relevant behavior scales, and neurocognitive assessments. Participants then used the program 5 times per day, 5 days a week for 4 weeks at home. At the postintervention visit, we collected the same data as during the preintervention visit and gathered additional feedback on their experience over the 4 weeks.

**Results:**

A total of 19 participants were included in the statistical analysis, showing significant decreases in scores across various categories. These included the Korean ADHD Rating Scale (Total: *P*=.004; Inattentive: *P*=.004; and Hyperactive-impulsive: *P*=.01) and Korean Conners’ Parent Rating Scale (Total: *P*<.001; Impulsive-hyperactive: *P*=.001; and Conduct Problem I: *P*=.04). Significant improvements were also noted in the Stroop word (*P*=.004), color (*P*<.001), and color-word (*P*<.001) scores. No significant differences in treatment effects were found between medicated and unmedicated participants. Caregiver and child satisfaction surveys yielded mean ratings of 4.3 and 4.1 out of 5, respectively.

**Conclusions:**

A 4-week gamified intervention improves attention and hyperactivity-impulsivity in children with ADHD, irrespective of medication status, demonstrating its potential effectiveness and acceptability as a treatment option. As this is a small pilot study and underpowered, larger studies with appropriate control groups are needed in future research.

## Introduction

Attention-deficit/hyperactivity disorder (ADHD) is a neurodevelopmental disorder characterized by persistent hyperactivity, impulsivity, or inattention that disrupts daily functioning [1]. The condition is heterogeneous, typically characterized by persistent patterns of inattention, hyperactivity/impulsivity, or a combination of both [2]. Most diagnoses occur early in life, with estimates indicating that approximately 3%-7% of children globally are diagnosed with ADHD [3,4]. Both in Korea and internationally, there has been a steady increase in ADHD diagnoses, primarily among children and adolescents [5,6].

In addition to the hallmark symptoms of persistent inattention and hyperactivity, ADHD can lead to cognitive and functional impairments in affected children, including challenges with response inhibition and working memory [2]. It also results in diminished academic performance and strained peer or family relationships, adding further distress to those affected [7]. Moreover, if not addressed, childhood ADHD can persist into adulthood, continuing to disrupt everyday life [8-10].

Child ADHD management strategies are categorized into pharmacological and nonpharmacological approaches. Although prescribed medication is frequently used to alleviate ADHD symptoms [10], parents often hesitate to medicate their children due to potential or actual side effects. These side effects commonly include decreased appetite, sleep disturbances, and stomach problems [4]. Furthermore, selecting the optimal medication for a particular patient can be time-consuming [10,11]. Nonpharmacological approaches include parental training, cognitive behavioral therapy, and cognitive training. These treatments can burden parents because they are both time-consuming and costly [12]. In addition, the scarcity of professional trainers makes access to nonpharmacological ADHD care more challenging. These limitations of traditional treatments have spurred the development of new ADHD treatments using advanced digital technologies.

Digital therapeutics (DTx) are defined as health software intended to treat or alleviate a disease, disorder, condition, or injury by generating and delivering a medical intervention that has a demonstrable positive therapeutic impact on a patient’s health [13-15]. They offer high accessibility in terms of both time and location [16] and considerably reduce concerns about side effects due to their noninvasive nature. Moreover, it demands less supervisory effort than other behavioral therapies, since embedded algorithms can automatically adjust the usage instructions and program difficulty according to the user's level.

Gamification is an additional strategy used by DTx to enhance user engagement and experience [17,18]. Given that children with ADHD often display heightened novelty-seeking behaviors [19], they generally favor game-based activities. Presenting therapeutic interventions in a gamified format can greatly improve adherence to treatment regimens.

Previous research has consistently shown that various multitasking tasks can mitigate ADHD symptoms [20,21]. However, the widely used dual n-back task is recognized for its high level of difficulty and complex rules. These characteristics can make onboarding challenging for children—particularly those with ADHD—leading to early dropout, and sustaining motivation for continued training can be difficult. Given these challenges of conventional interventions, some researchers developed a gamified intervention as an alternative treatment. One of the most notable approaches was EndeavorRx, a Food and Drug Administration (FDA)-cleared DTx, developed by Akili Interactive for children with ADHD, which is effective in mitigating inattentive symptoms [21-23]. It was developed by incorporating gamified cognitive training that involved multitasking Go/No-Go tasks and sensory-motor tasks [24]. The results showed that digital-based multitasking tasks, focusing on perceptual discrimination and sensory-motor coordination, were effective in enhancing attention in both older adults and children with ADHD [21,24].

Notably, the driving task effectively increases cognitive load while maintaining an engaging experience for children. Nevertheless, this type of multitasking approach also presents limitations, particularly in providing finely tuned difficulty adjustments. By implementing a difficulty adjustment system, it is possible to engage a broader range of age groups at different stages of cognitive development. This approach also helps address cultural differences related to disparities in access to digital technologies.

Therefore, we present a gamified DTx named “StarRuckus” (eMotiv). It aims to reduce core ADHD symptoms through the simultaneous execution of n-back and driving tasks, including a personalized difficulty adjustment algorithm to ensure training benefits [21,25,26]. It functions as an enhanced version of the Go/No-Go task (0-back task), allowing for the customization of difficulty levels through varying n levels [27,28]. While difficulty adjustment in Go/No-Go tasks typically involves manipulating factors such as stimulus presentation time, the number of stimuli, and the number of discriminative targets, the n-back task allows for additional parameters, including the n level and the inclusion of lure stimuli.

To the best of our knowledge, this study is the first to implement multitasking that combines an n-back task with a driving task as a cognitive training intervention aimed at improving ADHD symptoms. Computerized multitasking working memory training is recognized for its effectiveness in alleviating core symptoms of ADHD, such as inattention [29] and hyperactivity-impulsivity [20]. Multitasking n-back training strengthens the right inferior frontal gyrus, an area exhibiting structural [30,31] and functional deficits in children with ADHD [32,33]. Furthermore, multitasking driving training can enhance neural activity efficiency in the prefrontal cortex [24,34], thereby improving attention in children with ADHD [21,26,35].

Another distinctive feature of StarRuckus is its focus on the reward system within its gamification elements, setting it apart from other DTx interventions. After providing a personalized difficulty level tailored to each user, the system delivers differentiated in-game rewards based on performance outcomes. These rewards can be collected and used to unlock additional content, such as character purchasing and town customization.

This pilot study explored the effectiveness of StarRuckus as a potential treatment for children with ADHD, both medicated and unmedicated, while assessing its appeal to both children and their caregivers. We evaluated improvements in symptomatology and behavior scales as well as cognitive-neurological indicators before and after a 4-week intervention with StarRuckus. In addition, feedback on usage experience and safety was gathered from children and caregivers. Through this study, we aimed to provide preliminary evidence supporting the efficacy and acceptability of StarRuckus as a DTx.

## Methods

### Participants

Two cohorts, 1 taking medication and 1 without medication, were included in the study. The participants in both cohorts agreed not to alter their medication status during the study, understanding that any change could result in their withdrawal. Although this agreement was not binding, participants were permitted to change their medication status after consulting with their doctors. They were also required to report any medication changes during the study. Inclusion criteria for the participants consisted of (1) a confirmed *DSM-5* diagnosis of ADHD; (2) age between 6 and 12 years; and (3) either consistently taking medication for ADHD for at least 30 days or not taking psychotropic medication for at least 3 days to allow for a washout before baseline measures. Exclusion criteria for the participants included (1) current comorbid psychiatric diagnoses listed in *DSM-5* with marked symptoms; (2) initiation of behavior therapy within the last 4 weeks or plans to change or start behavior therapy during the study; (3) motor conditions reported by parents that prevent the use of digital interventions; (4) color blindness as reported by caregivers; (5) uncorrected visual or auditory impairments; and (6) any other medical condition that might confound the study data.

### Procedures

Participants were recruited from a community child center and a web-based community for caregivers of children diagnosed with neurodevelopmental disorders. Children and caregivers willing to participate completed a web-based survey to check eligibility. In addition, caregivers had to provide clinic-issued documentation as proof of their children’s medical histories. If children were eligible for the study, caregivers were contacted to provide instructions about the course of the study.

The study consisted of 3 phases: a preintervention visit, the intervention period, and a postintervention visit. At the preintervention visit, participants and their caregivers received detailed information about the research purpose, eligibility criteria, procedures, potential risks and benefits, and confidentiality aspects. After comprehending the study protocol, they provided informed consent. Subsequently, the caregiver and child were placed in separate rooms for data collection. While parents completed questionnaires regarding their children’s behavior, the children engaged in several neurocognitive tasks primarily focused on attention and working memory. Upon completion of the data collection tasks, children tested StarRuckus for approximately 10 minutes, with supervision to ensure compliance with the rules. At the conclusion of the preintervention period, tablets preinstalled with StarRuckus were lent to the participants for use during the intervention period. Throughout the intervention phase, participants had to complete 5 daily sessions, each lasting about 20-25 minutes, 5 days a week for 4 weeks. Compliance was electronically monitored by researchers, and reminder text messages were sent to parents when they were likely to fall short of the required session counts. In addition, caregivers and participants were instructed to report any adverse events to investigators in real time. The postintervention visit mirrored the preintervention visit, including neurocognitive tasks for the participants and questionnaires for the parents. Following data collection, satisfaction and inconvenience questionnaires were completed, incorporating reports of any adverse events. The children’s questionnaires were designed for readability, and researchers assisted the children with any difficulties.

### Digital Intervention

**Figure 1:**
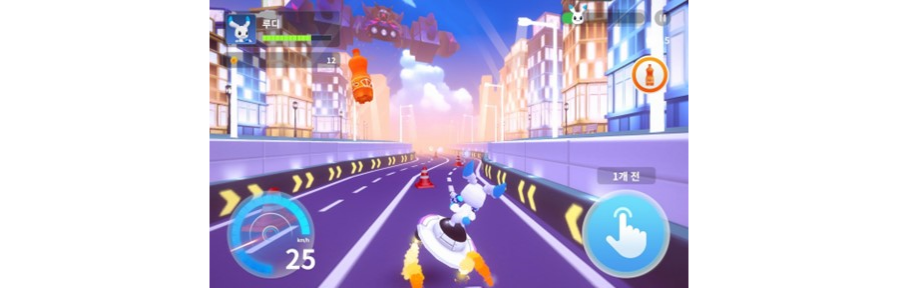
StarRuckus in-game screenshot.

StarRuckus (Figure 1) is a gamified training program designed for children aged 6 to 12. It is a digital application available for download on both Android (Google LLC) and iOS (Apple Inc) platforms, aimed at mitigating ADHD symptoms in children. The program includes two main tasks: (1) a continuous visuomotor driving task and (2) an n-back task [36], which engages working memory. In the driving task, users maneuver the character by tilting the device left or right. The primary objective of this task is to dodge as many obstacles as possible while collecting target points. The n-back task, a working memory exercise, challenges participants to identify whether each presented stimulus matches one shown several steps earlier in the sequence [37,38]. The maximum difficulty level in the app goes up to 4-back. Once all 5 sessions are completed, the training program ends automatically.

In a cognitive training program, it is crucial to maintain a level of difficulty that aligns with the users’ skill levels. The adaptive algorithm of StarRuckus adjusts task difficulty in real time based on individualized performance metrics tailored to each task type. For instance, in n-back tasks, increased difficulty may involve raising the n level or modifying stimulus parameters, such as increasing lure frequency or shortening stimulus duration. In driving tasks, difficulty can be modulated through speed changes. When users demonstrate improved performance, the system gradually introduces more challenging levels. Conversely, if performance declines, the difficulty is reduced to keep the tasks manageable and maintain engagement.

In addition to providing internal rewards through a gamified treatment designed to entertain children, the program included various in-app rewards to maintain participant engagement. For example, children were able to build their own villages with the resources gained during the training process. They could also use these resources to outfit new space vehicles and characters.

### Measures

#### Korean ADHD Rating Scale

A Korean ADHD Rating Scale (K-ADHD-RS) was used, originally developed by DuPaul in 1991 [39]. The K-ADHD-RS, comprising 18 questions, is a prevalent measure for assessing symptoms of ADHD. There are 2 subscales, 1 for inattention and 1 for hyperactivity. The Korean version, validated and proven reliable in 2002 [40,41], is extensively used in Korea to support ADHD diagnosis in children. In the scale, 0 corresponds to “rarely or never,” 1 to “somewhat,” 2 to “often,” and 3 to “always or very often.”

#### Korean Conners’ Parent Rating Scale

We used the Korean Conners’ Parent Rating Scale (K-CPRS), originally developed in 1970 [42,43]. It is a widely recognized tool for assessing problematic behaviors in children. Although analogous to the original, the Korean edition features a different distribution of questions across factors. According to the factor analysis results of the Korean edition [44], our assessment focused on 5 identified factors: Impulsive-hyperactive, Conduct Problem I, Conduct Problem II, Psychosomatic, and Anxiety [45].

#### ADHD Diagnostic System

The ADHD diagnostic system (ADS) is a computerized attention test designed to facilitate the diagnosis and assessment of treatment effects for ADHD. It is a type of continuous performance test [46,47] and features both ADS visual and auditory (ADS-A) modules, each containing 3 distinct stimuli (1 target and 2 nontargets). The proportion of targets was controlled to appear at 22% in the first part, 50% in the second part, and 78% in the final part of the task, respectively. Participants were instructed to press the spacebar when the target stimulus appeared and to ignore nontarget stimuli. Key outcome measures in our analyses included omission error, commission error, response time (RT), and RT SD [45].

#### Stroop Color and Word Test

In this study, we used the Korean version of the Stroop Color and Word Test [48], validated for children aged 5-14 years and originally developed in 1978 [49]. The Stroop test, primarily assessing selective attention and processing speed, consists of 3 card sessions: Word, Color, and Color-Word. Each session presents 100 stimuli, and children are timed to read them as quickly as possible within a 45-second limit. Specifically, children are instructed to read black words denoting color names (red, green, and blue) in the first session, name the printed color marked with “XXXX” in red, green, and blue in the second session, and name the printed color instead of the incongruently printed word in the final session. Raw scores from the word, color, and color-word sessions, along with the interference score, were recorded for data analysis.

#### Digit Span

The digit span (DS) test, a subcomponent of the Wechsler Intelligence Scale for Children-Fifth Edition [50], was administered using the Korean standardized version in this study [51]. The DS test, which involves memorizing a sequence of digits beginning with 2 digits and increasing by 1 digit at each step through 2 trials at each level until both are incorrect, was generally used to evaluate working memory and consists of 3 subtasks: DS Forward, DS Backward, and DS Sequencing. In the forward task, the examiner reads numbers aloud, and the participant recalls them in the order presented. The backward and sequencing tasks follow a similar procedure to the forward task but require participants to recall the sequence in reverse order or reorder the digits numerically, respectively. These tasks are generally more challenging than the forward task as they necessitate manipulating and reorganizing the received information. Scoring was based on the total number of successful trials across all subtasks.

### Statistical Analysis

Behavioral and cognitive outcome measures were analyzed using open-source statistical libraries in Python (version 3.9.12; Python Software Foundation). Depending on the Shapiro-Wilk normality test results, either a 1-sample *t* test (parametric) or a Wilcoxon rank-sum test (nonparametric) was performed. A 2-tailed significance level of *P*<.05 was considered statistically significant, and Cohen *d* effect sizes were calculated.

### Ethical Considerations

This study received approval from the Public Institutional Review Board designated by the Ministry of Health and Welfare, Korea (approval no P01-202305-01-007). Written informed consent was obtained from caregivers of all participating children, and assent was obtained from children when appropriate. Participants were informed about the study purpose, procedures, potential risks, and their right to withdraw at any time without penalty.

## Results

### Overview

The study is reported in accordance with the CONSORT-EHEALTH (Consolidated Standards of Reporting Trials of Electronic and Mobile HEalth Applications and onLine TeleHealth) checklist (Multimedia Appendix 1).

### Participants Flow

**Figure 2:**
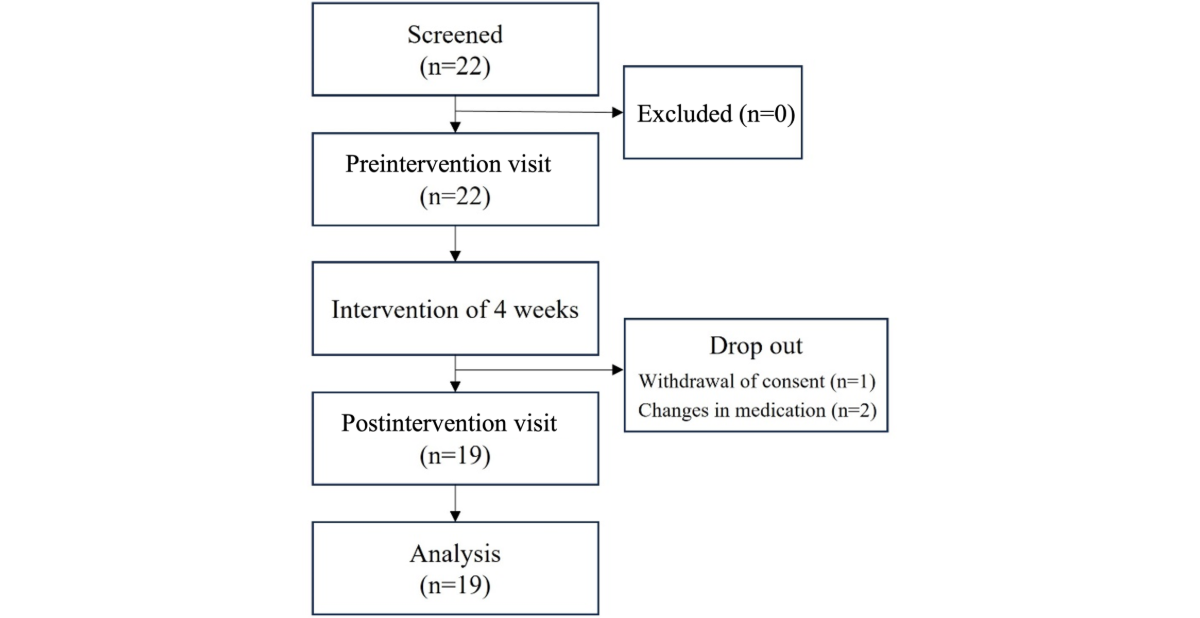
Flowchart of participants.

A total of 22 Korean participants were enrolled in the study following screening. During the intervention, 1 participant withdrew with parental consent. Two participants experienced changes in their medication status: 1 resumed medication during the study after not taking any at the preassessment, and another changed their medication type. A total of 19 participants completed the study, as shown in the participant flowchart (Figure 2). Table 1 presents the demographic and clinical characteristics of the participants at baseline.

**Table 1:** Demographic and clinical characteristics of the participants.

Characteristic			Count
**Sex** **, n (%)**			
	Male	14 (73.7)	
	Female	5 (26.3)	
Age (years), mean (SD)			8.5 (1.5)
IQ^a^, mean (SD)			99.4 (16.7)
**Medication, n (%)**			
	On medication	10 (52.6)	
	No medication	9 (47.4)	

^a^IQ: intelligence quotient.

### Parent Rating Scales

#### K-ADHD-RS

For the K-ADHD-RS, an ADHD symptom measure rated by parents, a paired *t* test assessed changes in the Total, Inattentive, and Hyperactive-impulsive scores before and after 4 weeks of intervention. There were statistically significant reductions in Total (*P*=.004; effect size *d*=0.69, 95% CI –9.27 to –1.99), Inattentive (*P*=.004; effect size *d*=0.77, 95% CI –5.27 to –1.15), and Hyperactive-impulsive scores (*P*=.01; effect size *d*=0.47, 95% CI –4.22 to –0.62; Table 2).

**Table 2:** Changes in attention-deficit/hyperactivity disorder (ADHD)-related symptoms and challenging behaviors of participants following the digital intervention.

Measure and factor			Pre mean (SD)		Post mean (SD)		*P* value		Effect size *d*
**K-ADHD-RS^a^**									
	Total	28.00 (8.78)		22.37 (7.55)		.004^c^		0.69	
	Inattentive	15.74 (4.45)		12.53 (3.84)		.004^c^		0.77	
	Hyperactive-impulsive	12.26 (5.42)		9.84 (4.79)		.01^c^		0.47	
**K-CPRS^b^**									
	Total	43.84 (14.57)		34.79 (10.34)		<.001^c^		0.72	
	Impulsive-hyperactive	14.00 (5.94)		10.26 (4.61)		.001^d^		0.70	
	Conduct problem I	9.00 (4.05)		7.53 (3.27)		.04^c^		0.40	
	Conduct problem II	0.21 (0.52)		0.26 (0.55)		.71^d^		-0.10	
	Psychosomatic	2.95 (2.50)		2.00 (2.34)		.15^c^		0.39	
	Anxiety	8.42 (3.76)		7.26 (3.11)		.06^d^		0.34	

^a^K-ADHS-RS: Korean ADHD Rating Scale.

^b^K-CPRS: Korean Conners’ Parent Rating Scale.

^c^*P* value derived from the *t* test.

^d^*P* value derived from the Wilcoxon signed-rank test.

#### K-CPRS

Postintervention changes in the Total score and the Impulsive-hyperactive, Conduct Problem I, Conduct Problem II, Psychosomatic, and Anxiety subscales from the K-CPRS, which assesses childhood behavior problems, were analyzed. Significant improvements were observed in Total (*P*<.001; effect size *d*=0.72, 95% CI –13.84 to –4.26), Impulsive-hyperactive (*P*=.001; effect size *d*=0.70, 95% CI –5.68 to –1.80), and Conduct Problem I (*P*=.04; effect size *d*=0.40, 95% CI –2.90 to –0.05) scales (Table 2).

### Neurocognitive Tests

#### ADS

For the ADS in the visual and auditory domains, postintervention changes—including omission error, commission error, mean of correct RT, and SD of correct RT for both visual and auditory tasks—were analyzed. Apart from a decrease in auditory commission error, which approached significance (*P*=.05; effect size *d*= 0.59), no other statistically significant or notable changes were observed (Table 3).

**Table 3:** Changes in neurocognitive test performances of participants with ADHD after the digital intervention.

Measure and factor		Pre mean (SD)	Post mean (SD)	*P* value	Effect size *d*
**ADS^a^-visual**					
	Omission error	0.05 (0.08)	0.09 (0.13)	.35^b^	–0.34
	Commission error	0.08 (0.12)	0.06 (0.07)	.42^b^	0.26
	RT^c^ mean	0.61 (0.28)	0.64 (0.29)	.71^b^	–0.10
	RT SD	0.22 (0.11)	0.25 (0.15)	.34^d^	–0.20
**ADS^a^-auditory**					
	Omission error	0.25 (0.17)	0.30 (0.22)	.60^b^	–0.26
	Commission error	0.16 (0.10)	0.10 (0.09)	.05^d^	0.59
	RT mean	1.11 (0.17)	1.18 (0.09)	.06^d^	–0.53
	RT SD	0.31 (0.10)	0.32 (0.14)	.28^d^	–0.15
**Stroop Color-Word Test^e^**					
	Word	50.72 (14.07)	56.94 (13.87)	.004^d^	–0.45
	Color	38.78 (10.00)	43.28 (10.14)	<.001^d^	–0.45
	Color-Word	21.33 (8.13)	25.11 (8.77)	<.001^d^	–0.45
	Interference	17.44 (6.40)	18.17 (3.99)	.60^d^	–0.14
**DS^f^**					
	Total	25.42 (6.53)	24.11 (6.70)	.32^b^	0.20

^a^ADS: ADHD diagnostic system.

^b^*P* value derived from the Wilcoxon signed-rank test.

^c^RT: response time.

^d^*P* value derived from a *t* test.

^e^In the case of the Stroop Color Word Test, statistical analysis excluded a participant who refused to undergo the test.

^f^DS: digit span.

#### Stroop Color and Word Test

Scores for the Word, Color, and Color-Word tasks, as well as the Interference score, were evaluated. One participant who did not complete the Stroop task on the postintervention visit was excluded from analysis. Scores for Word (*P*=.004; effect size *d*=-0.45, 95% CI 2.24-10.21), Color (*P*<.001; effect size *d*=-0.45, 95% CI 2.52-6.48), and Color-Word (*P*<.001; effect size *d*=-0.45, 95% CI 1.87-5.68) exhibited significant improvements. The Interference score did not show a statistically significant change (Table 3).

#### DS

In the WISC-DS, total scores for DS Forward, Backward, and Sequencing were analyzed. No statistically significant changes were noted in the DS tasks (Table 3).

### Medication Status Subgroup Analysis

As an exploratory analysis, we compared changes in outcome measures between participants taking medication (n=10) and those not taking medication (n=9). Independent *t* tests (or Mann-Whitney U tests, as appropriate) revealed no statistically significant differences in treatment effects between the medicated and unmedicated groups across all outcome variables. These findings suggest that the observed benefits of the digital intervention were independent of concurrent pharmacological treatment.

### Satisfaction and Acceptance

#### Satisfaction and Acceptance Reported by Children

The mean compliance score of the study participants during the intervention, calculated as the proportion of completed sessions to the total number of instructed sessions, was 81.8% (SD 21.8). For this calculation, only fully completed sessions were counted as compliant. For example, if a participant was instructed to complete 5 sessions per day, 5 days a week, for 4 weeks (totaling 100 sessions) and completed 80 full sessions, their compliance rate was calculated as 80%. Partial completions were not counted in the compliance score.

Participants completed an 11-item questionnaire assessing the digital intervention program's difficulty, learnability, enjoyment, likability, and desire for more play at the postintervention visit. The overall average rating was 4.3 on a 5-point Likert scale (1=strongly disagree, 2=disagree, 3=neutral, 4=agree, and 5=strongly agree). The mean ratings for task difficulty and learnability were 3.7 (SD 0.9) and 4.7 (SD 0.6), respectively. Enjoyment had a mean rating of 3.8 (SD 1.4), while the average likability for narrative structures, rewards, and sound effects was 4.1 (SD 0.9). The desire for more play received a mean rating of 3.9 (SD 1.2). Refer to Table 4 for additional results.

**Table 4:** Children’s ratings of satisfaction and acceptance with the digital intervention.

Dimension and question		Mean (SD)
**Difficulty**		
	Found the game easy to play.	3.7 (0.9)
**Learnability**		
	Understood the gameplay mechanics.	4.7 (0.6)
**Enjoyment**		
	Enjoyed playing the game.	3.8 (1.4)
**Likability**		
	Liked the appearance of the characters.	4.1 (1.2)
	Desired to collect the characters in the game.	4.5 (0.6)
	Liked the appearance of the space vehicles.	4.0 (1.3)
	Desired to collect the space vehicles in the game.	3.8 (1.3)
	Appreciated the game’s storyline	3.9 (1.1)
	Appreciated the rewards obtained from playing the game.	4.7 (0.6)
	Enjoyed the sound effects in the game.	3.7 (1.2)
**Continuity**		
	Look forward to continuing the game.	3.9 (1.2)

#### Satisfaction and Acceptance Reported by Caregivers

At the postintervention visit, caregivers completed a survey about their intervention experience. The survey included four items: (1) the extent to which their children enjoyed playing StarRuckus, (2) the presence of perceived risks to their children, (3) their willingness to continue using the program, and (4) the program’s ease of use. On a 5-point Likert scale (1=strongly disagree, 2=disagree, 3=neutral, 4=agree, and 5=strongly agree), the mean rating for enjoyment was 4.0 (SD 1.2), for the absence of perceived risks was 4.4 (SD 0.8), for willingness to continue was 3.6 (SD 0.8), and for ease of use was 4.5 (SD 0.6). The overall mean rating across the 4 items was 4.1 (SD 0.9). In addition, caregivers reported whether children experienced discomfort while using StarRuckus. Reported issues included arm pain (n=2), emotional reactions (n=2), and eyestrain (n=1) as intervention-related discomforts. Children who experienced arm pain reported that the tablet felt heavy. Regarding emotional reactions, 1 child felt frustrated with increasing task difficulty, and another became upset due to unsatisfactory outcomes despite considerable effort in the game. The child with eyestrain reported occasional glare or continuous blinking during app usage.

## Discussion

### Principal Findings

This study aimed to examine the feasibility and acceptability of the digital intervention StarRuckus, which targets the reduction of ADHD symptoms. Data on symptom and behavior scales, neurocognitive indicators, and user experience feedback were collected. The results of K-ADHD-RS and K-CPRS parental reports demonstrated significant changes in total scores and some subscales compared with baseline, with a moderate effect size indicating symptom improvement and associated behavioral or functional difficulties in children with ADHD.

Although the study lacked a control group, the observed improvements in behavioral symptoms are consistent with findings from previous controlled trials of digital cognitive interventions, which reported similar benefits beyond placebo or practice effects [21,23,52]. These precedents support the interpretation that the improvements observed in this study may reflect genuine intervention-related changes rather than nonspecific factors alone.

Although most results from neurocognitive tasks (ADS visual, ADS-A, and Stroop test) showed no significant changes, 3 subtasks of the Stroop test exhibited a significant increase in performance. Also, the commission error of ADS-A showed a marginally significant tendency to decrease. Furthermore, safety, user convenience, and satisfaction were confirmed by questionnaires administered to parents and children during the postintervention period, with no major adverse events leading to dropout. These findings provide preliminary evidence that gamified digital interventions could be an effective and safe treatment option for children with ADHD, regardless of medication status.

In the context of cognitive training, the transfer effect, a phenomenon where the outcomes of one learning event influence another, is a principal issue [53]. These effects appear within the realm of cognitive functions, or they may extend to behavioral aspects or symptoms. The current results provide preliminary evidence of transfer effects on behavior or symptoms in ADHD, in contrast to neurocognitive outcomes. Concerning the subjective and behavioral outcomes based on parental reports, our findings support a reduction in both inattention and hyperactivity-impulsivity symptoms of ADHD. These results are consistent with previous studies. Such studies have shown that digitalized cognitive training, including game-based therapeutics, can reduce the behavioral symptoms of ADHD [52,54,55]. Moreover, improvements were noted in the Conduct Problem I subscale of K-CPRS following the use of StarRuckus. Since we used K-CPRS, which differs in item composition and subscale names compared with the original version, interpreting these results requires caution. The Conduct Problem I subscale of K-CPRS, concerning oppositional behaviors and bad habits [40], involves argumentative and defiant behaviors, fidgeting or squirming, emotional dysregulation, and sleep problems. These symptoms are prevalent in ADHD and frequently co-occur with oppositional defiant disorder [3,56]. Indeed, exploratory findings from previous studies have suggested that computer-assisted cognitive training may yield symptomatic improvements in oppositional defiant disorder or conduct disorder itself [57,58]. Other evidence indicates that the efficacy of such training in alleviating ADHD symptoms is not substantially influenced by the presence of comorbid conditions, such as disruptive behavior disorders [59]. However, since the information was gathered by nonblinded raters and there was no control group, we cannot eliminate the possibility that parental overestimation may have influenced the results. A randomized controlled trial conducted by a blinded rater is needed for further investigation.

The current results provide insufficient evidence of transfer effects within neurocognitive functions. Based on the literature on digitalized cognitive training that targets executive functioning in children with ADHD, these findings can be attributed to several factors. First, the results of this study may have been influenced by the low ADS scores at baseline, as suggested by the exploratory analysis, which showed that many participants’ ADS performances were below the clinical threshold. Among the 8 indicators yielded by ADS, 13 (68%) participants out of 19 had none or only 1 deficit. These findings may partly relate to the heterogeneity in the cognitive profiles of ADHD [60,61]. Therefore, considering the observed improvements in subjective and behavioral outcomes, it is essential to use multiple cognitive outcomes to identify the underlying cognitive functioning in heterogeneous ADHD participants. Similarly, a previous study demonstrating the efficacy of ADHD digital interventions included children whose Test of Variables of Attention and Attention Performance Index scores, targeted by the intervention, met the inclusion criteria at baseline and found significant improvement in Attention Performance Index scores after training [21]. Second, it is possible that our cognitive training did not result in transfer effects within cognitive abilities such as auditory working memory and interference control. There is currently no consensus on the transfer effects of multitasking training. Finally, due to the small sample size of this study, there may not have been sufficient power to yield significant results. Therefore, future research involving measures targeting various domains of neurocognitive functions, brain signals, brain imaging, or mediating research could further elucidate the mechanisms of transfer in multitasking training. In addition, future studies with larger sample sizes may be necessary.

Although the current results provide preliminary evidence of transfer effects on behavior or symptoms in ADHD, they offer insufficient evidence for transfer effects within neurocognitive functions. Nevertheless, previous research suggests that the multitasking working memory training mechanism in this study likely involves updating and dual processing, which are central executive components of working memory considered vital for behavioral transfer in ADHD. Furthermore, a marginally significant reduction in ADS commission errors and an increase in 3 Stroop Test subtasks may suggest enhanced inhibitory control and processing speed, indicating potential cognitive transfer effects. These gains in inhibitory control, a primary deficit in children with ADHD [62], align with findings from previous studies [20,37]. These findings are further supported by overlapping neural systems, which may explain how working memory training leads to improvements in inhibition [63,64]. In addition, although the corpus of research is limited, several studies demonstrate that working memory training enhances processing speed in ADHD [65,66]. In this study, relatively simple cognitive tasks were used, and test-retest intervals of several weeks were applied; thus, the influence of practice effects may have been reduced [67]. However, the 2 factors previously mentioned do not eliminate practice effects. Therefore, further research with control groups is essential to verify hypotheses related to inhibitory control and processing speed.

While the small sample size limited statistical power, exploratory subgroup analyses indicated no significant differences in treatment outcomes between medicated and unmedicated participants. This finding provides preliminary support for the feasibility of using DTx both as a standalone and as an adjunctive intervention. Nonetheless, adequately powered future studies with stratified randomization are needed to more conclusively evaluate differential effects by medication status.

To promote high treatment adherence among children with ADHD, StarRuckus was developed as a gamified digital therapy, incorporating elements designed to foster internal motivation and maintain engagement in therapeutic activities. Notably, children rated the affinity of game components (eg, characters, space vehicles, and storyline) highly in terms of satisfaction and acceptance. In addition, there was a strong correlation between the children’s enjoyment and their desire for continued use (Pearson *r*=0.74; *P*<.001). This finding suggests that children who enjoyed the program for 4 weeks were more inclined to continue playing. Consequently, the game format is likely to encourage sustained engagement with the treatment.

StarRuckus was designed to maximize the advantages of gaming while minimizing its disadvantages, such as addictiveness and violence. It incorporates a playtime restriction that limits daily usage to 5 sessions per day. In addition, the story and audiovisual components are free from violent content. Notably, the item indicating the absence of risk factors in the program received a high average score (mean 4.4, SD 1.2 on a 5-point scale). Furthermore, digital programs, through their convenient usability, minimize the effort required for parental guidance. In the case of StarRuckus, parents reported that the program was easy to operate (mean 4.5, SD 0.6 on a 5-point scale), while children indicated a strong understanding of the task rules (mean 4.7, SD 0.6 on a 5-point scale).

The caregivers’ willingness to continue using the program received relatively low scores (mean 3.6, SD 0.8 on a 5-point scale). A similar pattern emerged in a preceding study of a digital intervention for children with ADHD [35], where 80% of parents found the program valuable, yet 40% were hesitant to continue the intervention (approximately 37% of parents responded “neutral” or “disagree” when asked if they wanted their child to continue using the program in this study). Researchers hypothesized that this reluctance might stem from the program’s perceived difficulty. Our study conducted a correlation analysis between the caregivers’ desire to continue and the children’s reported enjoyment, revealing a negative correlation of –0.29, which was not statistically significant (Pearson *r*=–0.29; *P*=.22). To better understand why caregivers of children with ADHD report high satisfaction yet exhibit reluctance to continue use, future research should include structured interviews to explore their motivations and concerns more deeply.

Among the 7 of 19 (37%) caregivers who were “neutral” or “disagree” about continuing the use of the program, only 1 reported discomfort (eyestrain) during participation. This suggests that discomfort did not notably influence their decision to continue using the program. In addition, no participant dropped out of the study due to discomfort.

### Limitations

This study has several important limitations that should be considered when interpreting our findings. First, the study had a small sample size. Second, and perhaps most critically, we had no control group, which makes it difficult to definitively attribute the observed improvements to our intervention rather than to natural progression, placebo effects, practice effects, or other external factors. The unblinded nature of our assessments further introduces the possibility of subjective bias, particularly in parent-reported outcomes. The improvements observed in behavioral measures and cognitive tasks could be influenced by practice effects or heightened parental attention during the intervention period. To solidify our understanding of the intervention’s efficacy, additional randomized controlled trials with appropriate control conditions (eg, waitlist, placebo, or active comparator) and larger participant cohorts are essential, with outcomes assessed by blinded raters. Finally, the heterogeneity of ADHD severity was likely due to participants’ differing times of diagnosis. Therefore, future studies need to use cohorts with controlled heterogeneity, enabling researchers to determine which ADHD subpopulations are most likely to benefit from our intervention.

### Conclusions

This is the first study to demonstrate the potential of a gamified multitasking training program that combines the n-back task and the driving task to improve core symptoms in Korean children with ADHD. In this preliminary investigation, we assessed the feasibility and appeal of a gamified DTx for children with ADHD, considering both medicated and nonmedicated groups. In both cohorts, the 4-week intervention was associated with reductions in core ADHD symptoms, including inattention and hyperactivity-impulsivity, and received favorable user feedback. StarRuckus, the digital program that is not bound by time or location and is readily accessible at home, holds promising potential to serve as both a substitute for and a supplement to conventional ADHD treatment strategies. While the preliminary results we observed provide an initial foundation for further research into this approach, our study design, which includes a small sample size without a control group, limits conclusions about causality and generalizability. Future studies should implement robust randomized controlled trials with appropriate control conditions, larger and more diverse samples, blinded assessments, and longer follow-up periods to further validate these preliminary findings and reveal the underlying mechanisms of the intervention’s effectiveness.

## Appendix

Multimedia Appendix 1 CONSORT-eHEALTH checklist (V 1.6.1).

## Abbreviations

ADHD: attention-deficit/hyperactivity disorder
ADS: ADHD diagnostic system
ADS-A: ADS-auditory
CONSORT-EHEALTH: Consolidated Standards of Reporting Trials of Electronic and Mobile HEalth Applications and onLine TeleHealth
DS: digit span
DTx: digital therapeutics
FDA: Food and Drug Administration
K-ADHD-RS: Korean ADHD Rating Scale
K-CPRS: Korean Conners’ Parent Rating Scale
RT: response time
